# Shotgun metagenomics dataset of the core rhizo-microbiome of monoculture and soybean-precedent carrot

**DOI:** 10.1186/s12863-025-01320-7

**Published:** 2025-04-12

**Authors:** Olubukola Oluranti Babalola, Alaba Adewole Adebayo, Ben Jesuorsemwen Enagbonma

**Affiliations:** Food Security and Safety Focus Area, Faculty of Natural and Agricultural Science, North-West University, Private Box X2046, Mmabatho, 2735 North West Province South Africa

**Keywords:** Agricultural sustainability, Cropping systems, Functional diversity, Illumina sequencing, Microbial structure

## Abstract

**Objectives:**

Carrot is a significant vegetable crop contributing to agricultural diversity and food security, but less is known about the core microbiome associated with its rhizosphere. More so, the effect of preceding crop and cropping history on the composition and diversity of carrot rhizo-microbiome remains largely unknown. With shotgun metagenomics, the study unveils how cropping systems direct rhizo-microbiome structure and functions, previously limited by other methods.

**Data description:**

Metagenomic-DNA molecule was extracted from four replicates each (12 samples) of a distant bulk soil and the rhizosphere soils from monoculture and soybean-precedent carrots, with the Power soil® DNA Isolation kit. The DNA samples were subjected to Next Generation Sequencing using the Illumina Novaseq X Plus (PE 150) platform. Raw sequencing reads were assembled and annotated with MEGAHIT and LCA algorithms in MEGAN software respectively, before a quality control check was done with FASTP. CD-Hit was used to de-replicate the sequences and the removal of host genomic-DNA and contaminant sequences was done with Bowtie2. The clean sequence data, in FastQ files, were analyzed for taxonomic classification and functional diversity of the rhizosphere microbiome using the Micro_NR and KEGG database respectively. The findings provide insights into microbiome dynamics, with potential implications for sustainable agricultural practices.

## Objective

Carrot (*Daucus carota* L.) is among the most cultivated vegetable crops in the world, not only for its edibility but also for the health benefits associated with its nutritional composition. As a taproot crop, the rhizospheric microbiome of carrots contributes significantly to the growth and development of the crop. However, the community structure, abundance, and diverse functionality of the core microbiome inhabiting carrot rhizosphere across cropping systems – mono-cropping and crop-rotation systems are less reported. To our knowledge, only a few studies on carrot microbiome have dealt with the rhizosphere [1], while most others focused on the endophytes [2, 3], studies on the impact of cropping systems on rhizo-microbiome are largely scarce. Majorly, previous assessments of the rhizosphere of carrots were based on culture-dependent and Sanger methods which offer inadequate information. These techniques are limited in unraveling the abundance of diverse functional genes, essential for unique metabolic pathways of agricultural importance. Therefore, this study presents a shotgun metagenomics dataset on the rhizosphere microbiome of monoculture and soybean-precedent carrot plants to better understand the microbial and functional shifts caused by the cropping systems with implications for sustainable agriculture. Data from this study will enable in-depth investigations into the potential of these microbes in improving soil fertility and health and enhancing the growth, yield, and productivity of plants – promising food security.

## Data description

The dataset consists of the raw Next Generation Sequencing (NGS) data from a shotgun metagenomics sequencing of DNA molecules extracted from the rhizosphere soil of monoculture (MCR) and soybean-precedent carrot (SCR), and a distant bulk soil (BS) from a farm in Gauteng, South Africa Gauteng Province, South Africa (26°06’21.0” S 27°33’35.5” E; 26°06’21.8” S 27°33’41.8” E; and 26°06’21.1” S 27°33’36.9” E GPS location respectively).

### Sampling

Four replicate soil samples at 0–15 cm depth were obtained from each sampling field. To capture a representative snapshot of the core microbial structure and functions, rhizosphere soils were sampled within the root development stage (specifically 50 days after sowing). The bulk soils were obtained from uncultivated fields approximately 10 m from planted fields. Samples were collected into sterile plastic bags with a hand trowel pre-sterilized with 70% ethanol before each sampling, and transported in a cooler box to the laboratory [4]. Samples were sieved (with a 2 mm sieve) [5], and stored in the refrigerator at − 20^o^C before analysis [6].

### Meta-DNA extraction and sequence

Metagenomic DNA in the soil samples was isolated by following the step-by-step procedure of the extraction kit (DNeasy^®^ Power soil^®^ DNA Isolation kit; Qiagen, Germany). The quality and concentration of the DNA extract were determined with a Nanophotometer^®^ NP80 Touch (Implen, Germany). A shotgun metagenomic library was prepared after fragmenting into segments (~ 350 bp) using a Covaris ultrasonic disruptor, and the quality of the constructed library was assessed with the Advanced Analytical Technologies Incorporated (AATI) analysis. The Illumina Novaseq X Plus (PE 150) system (Novogene, Singapore) was used to sequence the quality library [7].

### Data processing

Poor or low-quality reads and adapters in the raw data were trimmed and filtered using the Fastp (v0.23.4) [8], while contaminations from the host genomic DNA were removed, using Bowtie2 (v2.5.4) alignment tool [9]. MEGAHIT (v1.1.2) was used to assemble the clean data [10], before ORF of the scaftig (> = 500 bp) of each sample was predicted from the MetaGeneMark (v2.10) platform [11], then, sequences with lengths less than 100 nt were removed. The data were demultiplexed with CD-Hit (v4.6.8) [12] to achieve unigene sequences. Information on the sequence data obtained from all the soil samples is presented in Data file 1. The clean data (in FastQ files) were deposited on the Sequence Read Archive of the NCBI database with accession-ID numbers; SRP539180 (Data set 1), SRP537154 (Data set 2), and SRP537537 (Data set 3) for MCR, SCR, and BS respectively. Notably, there is no other data relating to this project published earlier or elsewhere. However, the project is ongoing with core aim of exploring this metadata with implication for sustainable agriculture and food safety.

Further analysis on taxonomic classification and functional diversity through alignment of these unigene sequences was performed in DIAMOND (v2.1.9) with parameter settings: blastp, -e 1e- 5, using Micro_NR and KEGG databases respectively [13]. The Lowest Common Ancestor (LCA) algorithm in MEGAN (v6.8.20) was used to annotate the sequences [14]. Overview of the community structure and diverse functionality of the rhizo-microbiome in this study are represented in Data file 2 respectively (Table 1).

**Table 1:** Overview of data files/data sets

Label	Name of data file/data set	File types (file extension)	Data repository and identifier (DOI or accession number)
Data file 1	General information on dataset	MS Excel file (.xlsx)	Figshare (10.6084/m9.figshare.28357343) [15]
Data file 2	Overview of microbial community structure and diverse functions from dataset	PDF file (.pdf)	Figshare (10.6084/m9.figshare.28357871) [16]
Data set 1	Core microbiome of monoculture carrot rhizosphere soil	FASTQ files (.fastq)	NCBI Sequence Read Archive (https://identifiers.org/ncbi/insdc.sra:SRP539180) [17]
Data set 2	Core microbiome of soybean-precedent carrot rhizosphere soil	FASTQ files (.fastq)	NCBI Sequence Read Archive (https://identifiers.org/ncbi/insdc.sra:SRP537154) [18]
Data set 3	Core microbiome of bulk soil from uncultivated field in a carrot farm	FASTQ files (.fastq)	NCBI Sequence Read Archive (https://identifiers.org/ncbi/insdc.sra:SRP537537) [19]

## Limitations

Not applicable. 

## Data Availability

The data described in this Data note can be freely and openly accessed on the NCBI SRA with accession-ID numbers; SRP539180 (Data set 1), SRP537154 (Data set 2), and SRP537537 (Data set 3), and the figshare databases under https://doi.org/10.6084/m9.figshare.28357343 (Data file 1); https://doi.org/10.6084/m9.figshare.28357871 (Data file 2). Please see Table 1 and references [15–19] for details and links to the data.

## Abbreviations

DNA: Deoxyribonucleic Acid
MCR: Monoculture carrot rhizosphere
SCR: Soybean-precedent carrot rhizosphere
BS: Bulk soil
GPS: Global Positioning System
NGS: Next Generation Sequencing
